# Interplay between Cellular Metabolism and Cytokine Responses during Viral Infection

**DOI:** 10.3390/v10100521

**Published:** 2018-09-24

**Authors:** Shu Zhang, Jessica Carriere, Xiaoxi Lin, Na Xie, Pinghui Feng

**Affiliations:** Section of Infection and Immunity, Herman Ostrow School of Dentistry, Norris Comprehensive Cancer Center, University of Southern California, Los Angeles, CA 90089-0641, USA; shu.zhang@usc.edu (S.Z.); jcarrier@usc.edu (J.C.); xiaoxil@usc.edu (X.L.); xnshina@gmail.com (N.X.)

**Keywords:** metabolic reprogramming, innate immunity, cytokine response, immunometabolism

## Abstract

Metabolism and immune responses are two fundamental biological processes that serve to protect hosts from viral infection. As obligate intracellular pathogens, viruses have evolved diverse strategies to activate metabolism, while inactivating immune responses to achieve maximal reproduction or persistence within their hosts. The two-way virus-host interaction with metabolism and immune responses choreograph cytokine production via reprogramming metabolism of infected cells/hosts. In return, cytokines can affect the metabolism of virus-infected and bystander cells to impede viral replication processes. This review aims to summarize our current understanding of the cross-talk between metabolic reprogramming and cytokine responses, and to highlight future potential research topics. Although the focus is placed on viral pathogens, relevant findings from other microbes are integrated to provide an overall picture, particularly when corresponding information on viral infection is lacking.

## 1. Introduction

Metabolic reprogramming of mammalian cells is part of adaptation to changes in the environment [1]. Recently, it has been recognized as an immediate defense response to infection by bacteria, viruses, and microbes [2,3,4]. The induction of innate immune responses demands significant metabolic resources, including energy, enzymes (e.g., kinases), and intermediates of macromolecular biosynthesis (e.g., transcription and translation) [5]. In addition, viral infection reprograms host metabolism and causes metabolic dysfunction, while hosts implement metabolic changes to mount effective defensive antiviral responses [1,5]. Findings from viral interference with cytokine regulation also advance our understanding of molecular mechanisms governing innate immune response [6]. Therefore, it is of great interest to elucidate the crosstalk between metabolism and cytokine responses.

The innate immune system is the front line of host defense. Upon infection, the innate immune system detects pathogen-associated molecular patterns (PAMPs) via pattern recognition receptors (PRRs), including Toll-like receptors (TLR), the retinoic-acid-inducible protein I (RIG-I)-like receptors (RLRs), Nod-like receptors (NLRs) and other cytosolic sensors (e.g., cGAS, IFI16, DAI, DDX41) [7,8,9]. Unlike bacterial pathogens, which are often sensed via lipopolysaccharides (LPS) and other cell wall components, viruses are frequently recognized by DNA, RNA, and glycoproteins. RIG-I and MDA5 sense double-stranded RNA (dsRNA) with 5′-triphosphate or di-phosphate, displaying specificity for dsRNA of distinct lengths or dsRNA derived from distinct viruses [10,11]. In addition, TLRs also sense diverse viral components. TLR3 detects viral dsRNA; TLR7 and TLR8 recognize viral single-stranded RNA (ssRNA); TLR9 senses viral DNA; and TLR2 and TLR4 respond to viral glycoproteins [12,13,14]. Through cognate adaptor molecules, PRRs activate two closely-related kinase complexes, IKK and TBK-1, that induce NF-κB and interferon regulatory factors (IRFs), respectively [15,16,17,18,19]. NF-κB and IRF collaborate with other transcription factors to up-regulate the expression and production of cytokines, all together constituting a potent antiviral response in mammals [20].

Immunometabolism is emerging as a new inter-discipline that integrates and elucidates the interplay between host metabolism and immune responses [21]. Pathogen infection is a common denominator of both of these host processes. Thus, studies on the metabolism-inflammation circuit in the context of viral infection, can provide valuable insights into the regulation of host immunity and inform the development of innovative antiviral therapeutics. This review seeks to summarize the regulatory role of metabolic events in the immune system upon viral infection and vice versa, and to present imminent research questions yet to be answered.

## 2. Metabolic Reprogramming Regulates Cytokine Responses against Viral Infection

**Figure 1:**
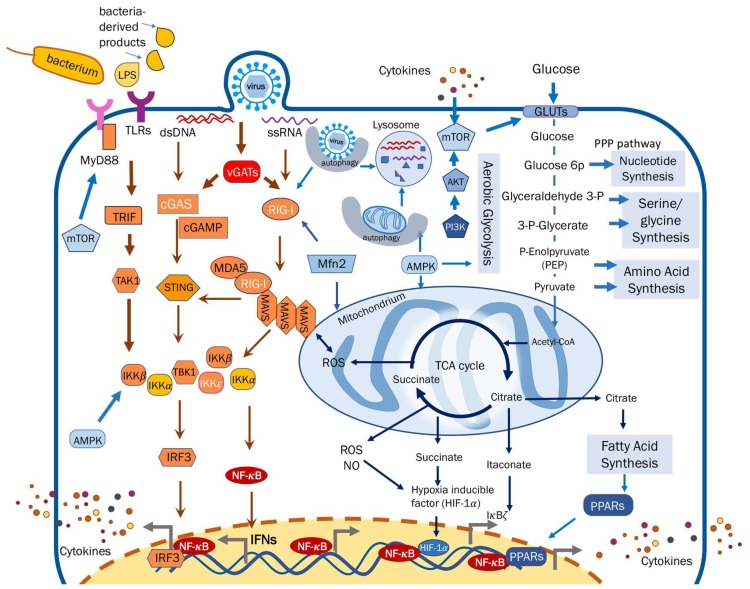
Schematic illustration of interplay between host metabolism and cytokine induction. Upon infection, pattern recognition receptors (such as Toll-like receptors (TLRs), retinoic-acid-inducible protein I (RIG-I)-like receptors (RLRs) and cytosolic sensors (cGAS)) sense pathogen-associated molecular pattern and dimerize with their cognate adaptor molecules to activate IKKαβ and TBK-1/IKKε. These kinases in turn activate IRF3 and NF-κB, thereby promoting cytokine production. Central metabolic pathways, including the glycolysis, tricarboxylic acid (TCA) cycle and lipid metabolism, are crucial for cell proliferation and viral replication. The immune-modulating effect of metabolism can stem from metabolites, metabolic enzymes and organelles. Conversely, the metabolism-programming activity of cytokine response can originate from immune signaling components or cytokine signaling thereof. Mitochondria serve as an excellent example that integrates diverse players at the interface of immune response and metabolism. Metabolites, such as succinate and citrate, directly or indirectly regulate NF-κB activation. Metabolic enzymes (e.g., mammalian target of rapamycin (mTOR) and adenosine 5′-monophosphate–activated protein kinase (AMPK)) play regulatory roles in both metabolism and immune responses. Metabolic organelles, such as mitochondria and lysosomes, deploy multiple strategies to interfere with pathogen replication and reprogram metabolic activity for intrinsic immune defense, via modulating mitochondrial fission and fusion (e.g., Mfn2) and activating the autophagy-lysosome degradative pathway. Blue arrows represent cellular metabolic processes and orange arrows indicate innate immune signaling pathways.

In general, infecting viral pathogens hijack and funnel the metabolic activity of host cells to favor viral replication, thereby disturbing the “normal” homeostasis of cellular metabolism [5,22]. Such a perturbation of host metabolism results in the alteration of intracellular metabolites and dysregulation of metabolic enzymes that can directly regulate or indirectly impinge on cellular immune responses (Figure 1).

### 2.1. Metabolites

#### 2.1.1. Carbohydrates

Citrate and succinate are two of major metabolic intermediates of the tricarboxylic acid (TCA) cycle and have a direct impact on the function of innate immune cells, such as macrophages [23]. Citrate and citrate carrier (CIC) are reported to accumulate in TLR4-activated macrophages [24], promote the production of prostaglandins, NO and reactive oxygen species (ROS), and depletion of CIC in activated macrophages reduces the production of these inflammatory mediators [25]. Succinate links the TCA cycle and mitochondrial respiration, and has similarly been recognized as an inflammatory signal that induces IL-1β production in a manner dependent on hypoxia-inducible factor-1α (HIF-1α) [24,26]. Furthermore, succinate dehydrogenase (SDH) can be inhibited by another mitochondrial metabolite, itaconate, in LPS-activated macrophages [27]. Itaconate and its membrane-permeable derivative dimethyl itaconate limit the production of inflammatory cytokines such as IL-1β, IL-18, IL-6 and IL-12, to curtail inflammation, providing a negative feedback for host immune responses [3,27]. These studies clearly indicate that metabolites of the TCA cycle can modulate cytokine production in host immune responses. However, it remains unknown whether succinate and citrate regulate immune response via similar mechanisms in viral infections.

Unlike viruses, most bacteria are extracellular pathogens that proliferate outside of host cells. Thus, bacterial structural components (e.g., LPS) and metabolites can induce immune responses and inflammation via cell surface receptors (e.g., TLR) or upon entering host cells in the form of secreted vesicles (e.g., exosomes), respectively [28,29]. A recent seminal study discovered that the bacteria-derived monosaccharide heptose-1,7-bisphosphate (HBP) activates the NF-κB signaling pathway to induce cytokine production [30]. Interestingly, alpha-kinase 1 (ALPK1) serves as a cytosolic innate immune receptor that directly senses ADP-β-d-manno-heptose (ADP-Hep), a metabolite of HBP, resulting in autophosphorylation and activation. Activated ALPK1 further phosphorylates TRAF-interacting protein with forkhead-associated domain (TIFA) and activates TRAF6-dependent NF-κB signaling in host cytosol, increasing the expression of IL-8 [31]. These findings establish a new paradigm whereby a microbial metabolite is sensed by a cellular kinase and pioneer a new research field that calls for further investigation.

#### 2.1.2. Lipids and Fatty Acids

One of the anabolic consequences of aerobic glycolysis in a viral infection is the inevitable increase in lipid biosynthesis, particularly with enveloped viruses. Clinical studies revealed that the serum concentration of low-density lipoproteins positively correlate with IL-6 in acquired immune deficiency syndrome (AIDS) patients infected with human immunodeficiency virus (HIV) [32]. Not surprisingly, IL-6 enhances lipid oxidation through an AMPK-dependent pathway in skeletal muscle [33], suggesting that there is a cross-talk between lipid metabolism and cytokines. In support of the crosstalk between lipid metabolism and inflammation, non-esterified fatty acids (NEFA) were reported to amplify cytokine secretion of TNF-α, IL-1β, IL-6 and IL-10 in human trophoblasts [34]. Unsaturated fatty acids, such as oleic acid, linoleic acid, and arachidonic acid, upregulate the production of IL-1α to aggravate inflammation in macrophages that are overloaded with cholesterol [35,36]. To reduce inflammatory responses, fatty acid oxidation, a major pathway that prevents the accumulation of fatty acids, can shift macrophage differentiation toward an anti-inflammatory state (M2), instead of a pro-inflammatory state (M1) [21]. In addition, lipid-activated nuclear receptor peroxisome proliferator-activated receptors (PPARs), such as PPAR-δ, were found to promote Janus kinase (JAK)-mediated phosphorylation of signal transducer and activator of transcription (STAT) proteins, and thereby further enhance the interferon (IFN) signaling in macrophages and B lymphoma cells [37,38]. Similarly, regulators of fatty acid synthesis, such as acetyl-CoA carboxylase and lysophosphatidic acid (LPA), influence the function of dendritic cells (DCs) and alter the production of cytokines, including IL-12, TNF-α and IL-10 [39,40]. However, the mechanistic detail of how these effectors control immune responses is still unknown.

#### 2.1.3. Amino Acids

Amino acids are major building blocks for cell proliferation and play important roles in controlling immune responses [21]. Glutamine is the most abundant amino acid in body fluid and cell culture medium, supplying energy and nitrogen for cellular metabolism. Glutamine enters the TCA cycle via the action of glutaminase (GLS) and glutamate dehydrogenase (GDH), and is an important alternative carbon source for virus-infected cells [5,41,42,43]. Initially, glutamine was found to augment cytokine responses and restrict microbial infection [44]. For example, the addition of glutamine to RAW264.7 macrophages stimulated with LPS elevated the production of IL-1α, IL-6, IL-10 and TNF-α [45]. Moreover, supplementing cultured T cells with glutamine up-regulated the expression of several IFN-γ inducible genes, which inhibited the reactivation of HSV-1 and HSV-2 [46]. These studies support the pro-inflammatory role of glutamine in immune responses by augmenting cytokine production. Surprisingly, in a human intestinal mucosa study, the addition of glutamine to duodenum tissue samples varied both pro- and anti-inflammatory immune responses. This is achieved via attenuating the production of IL-6 and IL-8, while increasing anti-inflammatory IL-10 [47]. Glutamine was later identified to regulate ubiquitin-conjugating enzymes, accelerating the ubiquitination and subsequent proteosomal degradation of IκBα [48]. This result further supports the conclusion that glutamine promotes NF-κB activation and inflammatory cytokine production. Findings from these studies further our understanding of glutamine’s physiological role in host immune responses and offer several examples that can be applied to studies of other amino acids and metabolites.

#### 2.1.4. Nucleotides

Besides the high demand for protein and lipid synthesis, viral replication also requires nucleotides and energy sources for synthesizing nucleic acids for transcription (messenger RNA) and translation (ribosomal and transfer RNA), viral genome replication (DNA) and generation of nucleotide adenosine triphosphate (ATP). ATP is the universal currency for energy in living cells, but is also a signaling molecule that engages the purinergic P2 receptor to trigger the production of several cytokines, including IL-12, IL-27, IL-13, IL-1β and IL-18 [49,50]. Similarly, a class of small non-coding RNA (miRNA) was also found to exert post-transcriptional regulation of gene expression and interfere with viral RNAs in plants, invertebrates and mammalian cells [51]. miRNA also regulates cytokine gene expression via inhibiting binding proteins of AU-rich elements (ARE) that are frequently found in regulatory regions of mRNA of inflammatory cytokines [52,53]. The recently discovered cyclic dinucleotide cGAMP, an endogenous nucleotide synthesized by cGAS upon sensing dsDNA in eukaryotic cells, serves as a secondary messenger that binds to stimulator of interferon genes (STING) and induces innate immune activation, culminating in inflammatory cytokine production [54]. STING also senses cyclic di-GMP and cyclic di-AMP, derived from bacterial pathogens, to induce IFNs [55,56]. In addition to regulating cytokine production, nucleotides can directly contribute to viral gene expression and genome replication by serving as targets sensed by PRRs. For example, RNase L, activated by 2′-5′ oligoadenylate synthase (OAS), cleaves viral RNA to block further viral replication and infection [57,58], while generating fragmented RNA to fuel RIG-I-dependent immune responses [59]. Moreover, nucleotide methylenecyclopropane analogs that can be phosphorylated by ppUL97 phosphotransferase, inhibit human cytomegalovirus DNA synthesis [60]. Nucleotide analogs such as ribavirin and acyclovir or its derivatives potently suppress the replication of diverse viruses [61]. These nucleotides can modify viral genetic materials allowing for the recognition of viral PRRs and activate the downstream signal transduction cascade leading to cytokine production.

### 2.2. Metabolic Enzymes

The complexity and plasticity of metabolism are the manifestation of the multi-functionality of metabolic enzymes. Mammalian target of rapamycin (mTOR) is a central metabolic regulator of immunity that integrates signaling events emanating from nutrient availability (e.g., amino acids) and growth factors [21,62,63]. It was also reported that mTOR interacts with MyD88 to activate IFN regulatory factor 5 and 7 (IRF5 and IRF7), thereby promoting cytokine production [64]. In addition, proteomics and bioinformatics analyses revealed a collection of mRNA-binding proteins implicated in regulating immune responses [65]. These RNA-binding proteins include many key metabolic enzymes, such as glyceraldehyde 3-phosphate dehydrogenase (GAPDH) of the glycolytic pathway; succinate-CoA ligase (SUCLG1) of the TCA cycle; and carbamoyl-phosphate synthetase 2, aspartate transcarbamylase, and dihydroorotase (CAD) of the de novo pyrimidine synthesis pathway [66,67]. For example, GAPDH can bind to the AU-rich elements within the 3′ untranslated region (UTR) of mRNAs of IFN-γ and IL-2 in CD4+ T cells [68]. The activation of glycolysis produces an excessive amount of glyceraldehyde 3-phosphate (G3P) that occupies GAPDH for oxidation, thereby preventing GAPDH from binding to IFN-γ and IL-2 mRNA to increase IFN-γ and IL-2 production. This study elucidates an elegant molecular mechanism wherein glycolysis enables T cell activation. Additionally, some DNA-binding proteins assume dual function in both metabolism and host immune responses. Nuclear hormone receptors (NHRs) are ligand-responsive transcription factors and regulators of mammalian lipid metabolism [69,70]. NHRs are also DNA-binding proteins and can interact with cytokine receptors to modulate the expression and secretion of cytokines, such as IL-2, IL-4, IL-6 and IL-7 [71]. However, the underlying molecular mechanism of the interaction between immune signaling transduction and the metabolic activity regulated by NHRs remains poorly understood and calls for further investigation.

Adenosine 5′-monophosphate-activated protein kinase (AMPK) is a metabolic regulatory enzyme that senses the intracellular concentration of ADP and AMP, and serves as a rheostat of cellular energy production [72]. A study on skeletal muscle regeneration reported impaired anti-inflammatory responses in macrophages in the absence of AMPKα1 [73]. In human endothelial cells stimulated with TNF-α, activated AMPKα2 phosphorylates IKKβ, which attenuates NF-κB activation [74]. Furthermore, AMPK directly phosphorylates JAK, thereby blocking activation of JAK-STAT signaling induced by IL-6 and constraining the pro-inflammatory response [75]. Although IKKβ and JAK are activated by autophosphorylation, their phosphorylation by AMPK appears to negatively regulate and temper inflammation. Another nutrient sensor, the aryl-hydrocarbon receptor (AhR), is also highly involved in innate immune signaling [76,77]. AhR is a ligand-activated transcription factor responsible for activating cytochrome P450, which degrades xenobiotics [78]. In response to influenza virus infection, AhR reprograms DCs’ differentiation, i.e., reducing CD103+ DCs and CD11b+ DCs in the lung-draining lymph node, while promoting virus-specific CD8+ T cells [79]. In CD4+ T cells, activated AhR increases the proliferation of TH17 cells that produce IL-17 and IL-22 [80]. Collectively, these studies indicate that AhR can promote immune cell differentiation to modulate cytokine production and program immune responses. A growing number of studies have unveiled new functions of metabolism-associated effectors in immune responses, which may pave the way to formulate new metabolic approaches to tailor antiviral immune responses.

#### Glutamine Amidotransferases and Deamidases

Glutamine amidotransferases (GATs) constitute a family of metabolic enzymes that catalyze the synthesis of nucleotides, amino acids, glycoproteins and enzyme cofactors [81]. Thus, GATs are key metabolic enzymes that are crucial for cell proliferation and, likely, viral replication. First reported more than half a century ago, protein deamidation is regarded as a non-enzymatic process that is associated with protein functional decay or “aging” in metazoans [82]. Studies on bacterial secreted effectors indicate that protein deamidation can be enzyme-catalyzed and highly regulated [83,84]. Investigating gamma herpesvirus’ immune evasion, our group discovered that gamma herpesvirus ORF75 proteins recruit cellular phosphoribosylformylglycinamidine synthetase (PFAS or FGARAT) to deamidate RIG-I, thereby preventing antiviral cytokine production [85,86]. Although they share homology with cellular PFAS, gamma herpesvirus ORF75 proteins lack the catalytic triad required for glutamine hydrolysis. Thus, they are referred to as viral GAT (vGAT) pseudoenzymes. The fact that gamma herpesviruses encode vGAT pseudoenzymes to hijack a metabolic PFAS for immune evasion is quite surprising and suggests that deamidation may provide an intrinsic link between metabolism and immune response. How these vGAT proteins specifically impact cellular nucleotide synthesis and generally affect metabolism remains unknown. In stark contrast to gamma herpesviruses, alpha herpesviruses express the UL37 tegument protein that functions as a bona fide deamidase during lytic replication [87]. UL37 can deamidate RIG-I and cGAS, thereby inactivating the innate immune signaling pathway provoked by both dsRNA and dsDNA [87,88]. Conversely, the deamidase-deficient HSV-1 more potently induces antiviral cytokines than wild-type HSV-1 in vivo and ex vivo. Whether UL37 participates or influences nucleotide synthesis or other metabolic reactions related to amidotransferase activity is an open question awaiting investigation. Nevertheless, these findings reveal a previously unrecognized enzyme activity and function of a metabolic glutamine amidotransferase in regulating innate immune responses, representing a potential link between metabolism and immune responses. Whether GAT-mediated protein deamidation is ubiquitous in metazoans needs further interrogation.

Besides their protein-deamidating activity, gamma herpesvirus ORF75 proteins were found to antagonize nuclear domain 10 (ND10)-mediated cell-intrinsic restriction, thus promoting viral lytic replication [89]. Specifically, ORF75 can induce the degradation of the ND10 component ATRX and relocate ND10 Sp100, dismantling the ND10 complex [89]. Whether ND10 is involved in cellular metabolism with or without viral infection would be intriguing to examine. Moreover, MHV68 encodes three vGAT homologues, including vGAT (ORF75c), ORF75b and ORF75a. vGAT protein deamidates RIG-I to evade antiviral cytokine production [90], while ORF75a promotes the early stage of viral replication and increases cell death by virtue of increasing TNF-α production [91]. Thus, viruses have evolved diverse means to evade immune responses via multifunctional viral proteins that provide molecular links between immune response and other cellular activities, such as metabolism.

### 2.3. Eukaryotic Organelles

#### 2.3.1. Mitochondria

Mitochondria are core organelles of biosynthesis and energy production in eukaryotic cells, but are also an essential compartment for regulating immune responses in mammalian cells [92]. For example, the oxidative phosphorylation (OXPHOS) activity of mitochondria is critical to activate RLR-mediated signal transduction and interferon production [93]. Damage-associated molecular patterns (DAMPs), similar to PAMPs, can initiate innate signaling to induce cytokines to repair cellular damage [94]. TLR9 and formyl peptide receptor-1 (FPR-1) recognize mitochondrial DNA and N-formyl peptides, respectively, to ultimately induce cytokine production [94]. In response to microbial infection, mitochondria generate reactive oxygen species (ROS) to kill microbes directly. Mitochondrial ROS (mtROS) can also serve as signaling molecules to further induce the production of pro-inflammatory cytokines (e.g., IL-6 and TNF-α) and the activation of pyrin domain-containing 3 (NLRP3) inflammasome that processes IL-1 for secretion [95,96]. Furthermore, mtROS can activate mitochondrial antiviral-signaling protein (MAVS) and its downstream pathways through cytochrome c oxidase (CcO) to produce IFNβ [97]. Apart from mtROS signaling, mitochondria also provide a membranous platform for MAVS to assemble signaling complexes relaying innate immune activation downstream of cytosolic dsRNA sensors RIG-I and MDA5 [98]. Upon activation by dsRNA, RIG-I interacts with and induces the oligomerization of MAVS to form prion-like aggregates [98]. MAVS aggregates recruit IKKα, IKKβand IKKε kinases via the C-terminal segment, leading to the activation of IRF3 and NF-κB [99,100,101,102].

Mitochondria are dynamic organelles, constantly undergoing fission and fusion to adapt to cellular metabolic needs or in response to environmental cues. Fission and fragmentation are promoted to eliminate damaged mitochondria and maintain normal metabolic function; whereas fusion and elongation enrich metabolites and energy sources (e.g., ATP) in the presence of external stressors, such as viral infection [103]. For example, Dengue virus infection induces mitochondrial elongation by the viral NS4B protein, which antagonizes the fission effector dynamin-related protein (Drp1) [104]. Elongated mitochondria reshape mitochondria-associated membranes (MAMs), which impairs RIG-I translocation to MAMs and further quenches innate immune responses [104]. Interestingly, hepatitis C virus (HCV) promotes mitochondrial fission by inducing phosphorylation of Drp1, resulting in its mitochondrial translocation to promote fragmentation. HCV-induced mitochondrial fission disrupts the interaction between MAVS and RIG-I, impairing IFN induction [105]. Influenza A viral protein PB1-F2 can translocate into the mitochondrial inner membrane through Tom40 channel, induces mitochondrial fission and further impairs the RIG-I-mediated interferon induction by decreasing the mitochondria membrane potential [106,107]. Furthermore, emerging evidence also revealed that mitochondrial mitofusin-2 (Mfn2) can directly interact with MAVS to fragment mitochondria, resulting in the inactivation of IRF-3 and NF-κB downstream of RIG-I [108,109]. Supporting the significance of mitochondria in innate defense against HCV, proteolytic cleavage of MAVS from the mitochondrial membrane potently diminishes RIG-I-mediated innate immune activation in hepatocytes infected with HCV [105,110]. Collectively, these studies demonstrate that mitochondria participate in a broad spectrum of immunological functions to restrict microbial invaders via the activation of MAVS-mediated immune pathways, production of ROS and modulation of fission and fusion dynamics.

#### 2.3.2. Lysosome

Lysosomes are the primary organelles responsible for the degradation of large protein aggregates or damaged organelles, thereby recycling amino acids, fatty acids and nucleotides to maintain cellular homeostasis [111]. Lysosomes can also regulate the induction of inflammatory cytokines in response to pathogen-associated molecules, such as microbial peptidoglycan and LPS [112]. The hydrolysis of peptidoglycan by lysosomes converts peptidoglycan into a “mature” form that is recognized by its cognate receptor, thus inducing TNF-α in monocytes and IL-8 in neutrophils [113]. The activity of lysosomes can impact viral infectivity directly. For example, the inhibition of lysosome greatly promotes HIV-1 infection due to the blockade of lysosomal degradation of HIV virions [114].

Autophagy, a lysosome-dependent degradative process, is an important cellular defense machinery to dispose of xenobiotic substances and intracellular microbes [115]. To limit viral replication, autophagy targets newly assembled viral particles, and processes viral nucleic acids to activate endosomal TLRs, initiating type I IFN immune responses [116]. Autophagy-related protein, ATG5, together with ATG12, bind to RIG-I and MAVS to prevent CARD-mediated signal transduction under physiological conditions, thereby limiting excessive inflammatory responses [115,117]. Indeed, loss of Atg5 in mouse embryonic fibroblasts (MEFs) increased the production of type I IFN in response to vesicular stomatitis virus, demonstrating negative regulation of antiviral responses by ATG5 [118]. Therefore, the autophagy-lysosome degradative pathway plays a pivotal role in balancing a sufficient, but not excessive, cellular inflammation.

## 3. Cytokine Responses Guiding Host Metabolic Activity

Cytokines are a group of signaling polypeptides that modulate a broad spectrum of biological processes via cell surface receptors [119]. Functionally, cytokines are classified into three groups: adaptive immunity, such as IL-2 and IL-4; pro-inflammatory signaling, including interferons (Type I, II, III), interleukins (e.g., IL-1, IL-6, TNF, IL-17); and anti-inflammatory signaling, such as IL-12 and IL-10 [120]. In response to external microbial infections or internal stress stimuli (e.g., cancer), host cells secrete cytokines to reprogram cellular metabolism as a defense mechanism. Although cytokines are traditionally studied in immune responses, the metabolism-modulating activity of cytokines is increasingly recognized [21]. Research into the versatile functions of cytokines and related signaling events can advance our understanding of immune regulation and related processes (such as metabolism), paving the way for the development of innovative therapeutics for infectious diseases.

The Warburg effect was initially defined in cancer cells. It is the metabolic shift from oxidative phosphorylation (OXPHOS) to glycolytic metabolism (aerobic glycolysis) even with sufficient oxygen supply, providing intermediates for macromolecule synthesis needed during cell proliferation [121,122]. Although the net ATP production efficiency of aerobic glycolysis is much lower than that of OXPHOS, the pentose phosphate pathway (PPP) and serine/glycine pathway shunt glycolytic intermediates to support the production of purines, pyrimidines and amino acids that are crucial for viral replication and cell proliferation [121]. In addition, recent studies have revealed several mechanisms employed by viruses to reprogram host metabolism to complete viral life cycles [5]. For instance, Kaposi’s sarcoma-associated herpesvirus (KSHV) utilizes miRNA to hinder mitochondrial biogenesis and facilitate the installment of an aerobic glycolysis program to establish and maintain viral latency [123]. Dengue virus (DENV) infection upregulates the expression of both glucose transporter 1 and hexokinase 2 to switch glucose metabolism to aerobic glycolysis [124]. Thus, viruses funnel cellular metabolic activity to favor their infection, but cells may still be able to revert these changes to restrict viral infection. Infection-induced cytokines indeed play a vital role in metabolic reprogramming, as summarized in Table 1.

Pro- and anti-inflammatory cytokines produce functionally opposing metabolism-modulating activities and consequences in response to microbial infection. Anti-inflammatory IL-10 was found to impede glycolysis switch by inducing an mTOR inhibitor, DDIT4, to prevent glucose uptake [132]. However, a recent report revealed that IL-2 increased glucose metabolism via the Akt-mTOR signaling pathway, promoting proliferation of T helper 1 (Th1) cells and enabling a Th1-skewed immune response [128]. Moreover, ATP production and lactate export are accelerated in response to TNF-α, glycolysis, which correlates with increased cellular glucose uptake through up-regulation of the expression of glucose transporter GLUT1 [125]. However, the effect of inflammatory cytokines on cellular metabolism is also context-dependent on the current physiological condition. For example, TNF-α is able to induce insulin resistance by antagonizing tyrosine phosphorylation of the insulin receptor and its major cytosolic substrate, insulin receptor substrate 1 (IRS-1) [126]. TNF-α and IL-6 were reported to suppress vitamin D metabolism in colonic epithelial cells by down-regulating the vitamin D-activating enzyme CYP27B1 [127]. Studies on obese individuals showed that IL-6 modulates glucose metabolism in myeloid cells and induces M2 polarization via up-regulating the expression of IL-4 receptor in macrophages, which further exacerbates insulin resistance [135,136]. IL-4 was also identified to promote the expression of glucose transporter 4 (GLUT4), enhancing lipogenesis and reducing lipolysis, which results in fat accumulation in mature rat adipocytes [129]. However, an opposite phenotype was reported in a similar study using 3T3-L1 pre-adipocytes [130], suggesting that anti-inflammatory IL-4 cytokine function may be tissue specific and reflect the metabolic plasticity.

Interferons are major antiviral cytokines secreted by diverse host cells in response to viral infection [137]. Type I IFNs can induce fatty acid oxidation in plasmacytoid dendritic cells by increasing the expression of PPAR-α [2]. In addition, IFN-mediated innate immune responses alter the lipid supply in macrophages by reducing intracellular lipid biosynthesis while promoting extracellular lipid import [134]. The decrease in lipid biosynthesis suppresses viral assembly as viral replication requires lipid levels that exceed normal cellular supply. Moreover, cholesterol 25-hydroxylase, identified as an antiviral interferon-stimulated gene (ISG), transforms cholesterol into 25-hydroxycholesterol, which restricts viral replication [138]. IFN-γ was found to inhibit the central metabolic regulator mTORC1 and repress mRNA translation to reshape macrophage metabolism in favor of host defense [139]. IRFs also play pivotal roles in regulating cellular metabolism. IRF3 inhibits the expression of retinoid X receptor α, leading to hepatotoxicity associated with viral infection [140]. Suppressor of cytokine signaling (SOCS) proteins are a group of inhibitors of cytokine signaling [141]. To prevent excessive inflammatory responses, SOCS1 negatively regulates a number of glycolytic enzymes, such as hexokinase, lactate dehydrogenase A and GLUT1, in macrophages during sepsis [142]. In this sepsis model, macrophage metabolic reprogramming is dependent on the STAT3/HIF1α/glycolysis axis and inhibition of glycolysis ameliorates susceptibility to sepsis. Hence, interferons and their effectors have indirect antiviral activity by reprogramming cellular metabolism. These findings on how cytokines govern the host metabolism advance our knowledge of the molecular link between immune response and metabolism during viral infection.

## 4. Perspectives

Metabolism is a complex and systematic biological process, comprising an enormous number of interconnected cellular biochemical reactions and signaling transduction pathways. Advances in immunology unveil the critical role of host metabolism in antimicrobial responses and of immune responses in metabolic reprogramming. Here, we highlight a few imminent questions in immunometabolism in the context of viral infection that may further illuminate the interaction of metabolism and cytokine responses.

### 4.1. Molecular Mechanism of Virus-Induced Metabolic Reprogramming

As viruses demand high metabolism to meet rapid viral replication, they likely deploy their gene products to reprogram host metabolism, while impeding host immune defense (e.g., the production of cytokines). Identification and functional characterization in immunometabolism of these viral gene products will likely elucidate new molecular mechanisms by which metabolism is programmed and immune response is regulated. The vGAT pseudoenzymes of gamma herpesviruses and their functional homologues of herpes simplex viruses provide a system in which innate immune activation and metabolism may be intrinsically coupled via protein deamidation [85,86,87], although the role of these viral enzymes in metabolic control remains to be determined. To date, a limited number of viral gene products have been implicated in modulating cell metabolism [5,22,143], while much more is known about viral immune manipulation. More importantly, these two processes are often independently studied in the context of viral infection. The growth of the interdisciplinary field of immunometabolism is expected to foster cross fertilization of these two subjects and unravel exciting molecular links between them.

### 4.2. Metabolic Reprogramming by Anti- and Pro-Inflammatory Cytokines

Viral infection can trigger the production of both anti- and pro-inflammatory cytokines, which systemically exert their effects on immune response and metabolism [144]. Even though our understanding of metabolic reprogramming by various cytokines in cultured cells is rapidly progressing, the ultimate goal remains to define the metabolic profile at the tissue, organ and body level during immune response against viral infection. Innovative systems, entailing novel engineered animal models and metabolite detection approaches or techniques, are urgently needed to monitor the dynamic changes of immune response and metabolites in real time. Clustered Regularly Interspaced Short Palindromic Repeats (CRISPR) technology and evolving imaging techniques may be leveraged in the future to fill this gap.

### 4.3. Immunometabolism in the Context of Inter-Kingdom Microbial Infections

Current studies of immunology mainly concentrate on either bacterial or viral infection on host cells, while co-infection studies with multiple species of the same kingdom or trans-kingdom are burgeoning [145]. The human microbiome contains bacteria, fungi and viruses, and this poly-microbial consortium likely render a distinctly different immune-metabolic profile than those endowed by any individual player [146]. Moreover, these pathogens may extensively collaborate to evade immune responses. For example, bacterial quorum-sensing molecules have been found to inhibit RIG-I induced antiviral innate immunity, which facilitates viral replication [147]. In return, viruses may play a role in bacteria- and fungi-induced immune signaling transduction, but this needs to be explored to further understand immune signaling pathways that interface between bacteria, viruses and mammalian cells.

## Figures and Tables

**Table 1:** Role of important cytokines in metabolism.

Cytokine	Effects on Metabolism	References
TNF-α	Induces Insulin resistance; increase glycolysis, adenosine triphosphate (ATP) production, and lactate export; reduce vitamin metabolism	[125,126,127]
IL-2	Increases glucose metabolism via Akt-mTOR signaling to promote T cell differentiation	[128]
IL-4	Up-regulates the expression of glucose transporter 4 (GLUT4); enhance glucose and lipid metabolism	[129,130]
IL-6	Reduces vitamin metabolism; enhance lipolysis	[127,131]
IL-10	Promotes insulin sensitivity; inhibits aerobic glycolysis and promotes oxidative phosphorylation.	[132,133]
IFNs	Induce fatty acid oxidation; reduce lipid biosynthesis	[2,134]
